# Understanding the Pharmacology of COVID-19 mRNA Vaccines: Playing Dice with the Spike?

**DOI:** 10.3390/ijms231810881

**Published:** 2022-09-17

**Authors:** Marco Cosentino, Franca Marino

**Affiliations:** Center for Research in Medical Pharmacology, University of Insubria, 21100 Varese, Italy

**Keywords:** COVID-19, mRNA vaccines, SARS-CoV-2, spike protein, pharmacology, safety, adverse effects, pharmacodynamics, pharmacokinetics

## Abstract

Coronavirus disease-19 (COVID-19) mRNA vaccines are the mainstays of mass vaccination campaigns in most Western countries. However, the emergency conditions in which their development took place made it impossible to fully characterize their effects and mechanism of action. Here, we summarize and discuss available evidence indicating that COVID-19 mRNA vaccines better reflect pharmaceutical drugs than conventional vaccines, as they do not contain antigens but an active SARS-CoV-2 S protein mRNA, representing at the same time an active principle and a prodrug, which upon intracellular translation results in the endogenous production of the SARS-CoV-2 S protein. Both vaccine-derived SARS-CoV-2 S protein mRNA and the resulting S protein exhibit a complex pharmacology and undergo systemic disposition. Defining COVID-19 mRNA vaccines as pharmaceutical drugs has straightforward implications for their pharmacodynamic, pharmacokinetic, clinical and post-marketing safety assessment. Only an accurate characterization of COVID-19 mRNA vaccines as pharmaceutical drugs will guarantee a safe, rational and individualized use of these products.

## 1. Introduction

In most Western countries, the mass Coronavirus disease-19 (COVID-19) vaccination campaigns, which have been ongoing since the end of 2020, are based on two mRNA vaccines against SARS-CoV-2 (BioNTech–Pfizer BNT162b2 and Moderna mRNA-1273) [1,2]. Both products contain mRNAs encoding the SARS-CoV-2 spike (S) protein, which is essential in the binding of the virus to the host cells expressing its receptor angiotensin-converting enzyme 2 (ACE2). These products were presented from the outset as intrinsically safe, since it was believed that, similar to conventional vaccines, after intramuscular injection, most of the dose would remain in the muscle and the rest would drain through the lymphatic system, being eventually captured by antigen-presenting cells and B cells and undergoing complete elimination in a few tens of hours at the most [3,4]. On this basis, the public was explicitly reassured by influential blogs (see for example [5]) as well as by academic institutional web pages (see for example [6]) that these products were not expected to exhibit any relevant systemic disposition and that the resulting S protein would remain attached to the surface of the cells and would not be released in the bloodstream and tissues to encounter ACE2 receptors and eventually induce organ damage. Step by step, however, it became clear that this was not the case.

## 2. Evidence for Systemic Biodistribution of COVID-19-Vaccine-Induced S Protein in Vaccinated Subjects

A study published in May 2021 documented for the first time circulating vaccine-induced S protein in the blood of 11 out of 13 subjects as early as one day after injection of the Moderna COVID-19 vaccine, up to 150 pg/mL and for about two weeks after injection [7]. Immediately, it was observed that such concentrations were several orders of magnitude lower than those needed to bind ACE2 receptors, and that in any case after two weeks no trace of the protein was detectable in blood. Soon after, however, a report was published describing the case of a woman suffering from Moderna-COVID-19-vaccine-induced thrombocytopenia and with 10 ng/mL vaccine-induced S protein levels in plasma 10 days after vaccination [8], thus nearly 100 times higher than those reported previously [7], suggesting excessive vaccine-induced production of S protein as a determinant of vaccine toxicity. Meanwhile, both vaccine mRNA and vaccine-induced S protein were shown in axillary lymph nodes up to 60 days after the second dose of both Moderna or BioNTech–Pfizer COVID-19 vaccines [9], thus showing that endogenous production of S protein following vaccination may occur for much longer than previously thought. The S protein has been so far identified in endomyocardial biopsies of patients with myocarditis up to nearly two months following COVID-19 vaccination [10], in circulating monocytes of patients with post-acute sequelae of COVID-19 (PASC)-like symptoms following COVID-19 vaccines [11], in the vesicular keratinocytes and endothelial cells in the dermis in a patient who had persistent skin lesions due to varicella zoster virus (VZV) reactivation over three months after COVID-19 vaccination [12], and the S protein mRNA was present in the right deltoid and quadriceps muscles of a woman with myositis one month after injection of the BioNTech–Pfizer COVID-19 mRNA vaccine into the left deltoid muscle [13]. Taken as a whole, evidence strongly supports the possible link between inappropriate expression of S protein in sensitive tissues and subsequent tissue damage.

## 3. Adverse Effects Following COVID-19 Vaccination: Too Much S Protein, for Too Long and/or in the WRONG Place?

A comprehensive review of the literature recently discussed the role of COVID-19-mRNA-vaccine-induced S protein in adverse effects following vaccination [14], and we showed that production of S protein induced by COVID-19 mRNA vaccines may well compare to the estimated production during SARS-CoV-2 infection [15]. The present opinion paper identifies, develops and discusses the implications of the role of the S protein in adverse effects following vaccination and indicates the most appropriate pharmacological approaches for a better characterization of these vaccines, with the aim of providing guidance towards their rational and individualized use. Indeed, based on these premises, a major explanation of adverse effects following COVID-19 vaccination could well be that mRNA vaccines induce in selected individuals excessive production of S protein, for too long and/or in inappropriate tissues and organs, and this occurrence is at present unpredictable, since systemic biodistribution and disposition of the COVID-19 mRNA vaccine has so far never been considered an issue, and as a consequence it has never been studied as it would have actually deserved. Remarkably, the inadequate understanding of how to target specific organs and cells for protein expression is well-acknowledged as one of the major limitations of mRNA gene therapy [16]; however, for mRNA vaccines, it has been so far ignored.

## 4. COVID-19 mRNA Vaccines: Pharmaceutical Drugs Rather than Conventional Vaccines

In other words, considering COVID-19 mRNA vaccines the same as simple conventional vaccines was a major misunderstanding, since they are quite distinct and in specific ways better reflect pharmaceutical drugs and should be therefore considered as such. COVID-19 mRNA vaccines contain active SARS-CoV-2 S protein mRNA, which represents at the same time a prodrug and an active principle. Although it might sound unconventional to define the content of a vaccine as a prodrug, the definition undoubtedly applies to these products, which are also unconventional in general, given their completely innovative conception, which even required updating the meaning of the word “vaccine” in vocabularies (see for example the Merriam-Webster Dictionary [17]). As such, these products urgently need a proper conceptualization. Conventional vaccines contain antigen(s), which represent their active component, in turn exerting their effect by acting on endogenous targets (the immune system cells). On the contrary, mRNA vaccines do contain a molecule (the mRNA) which is unable to trigger any anti-SARS-CoV-2 immune response unless it is translated by endogenous cell metabolism into an active moiety, which is the viral S protein. In other terms, mRNAs contained in vaccines fully meet the definition of a “prodrug” as reported, for example, in the Merriam-Webster Dictionary: “*a pharmacologically inactive substance that is converted in the body (as by enzymatic action) into a pharmacologically active drug*” [18], which is the case for vaccine-derived mRNA, converted into active S protein by ribosomes through their catalytic peptidyl transferase activity that links amino acids together, leading to protein synthesis. According to the conventional classification of prodrugs [19], COVID-19 mRNA vaccines could be classified as type I prodrugs since they undergo intracellular conversion. The tissue location where conversion occurs is, however, uncertain, since the catalytic mechanism leading to protein synthesis is common to all the cells in any tissues and organs, with the notable exception of erythrocytes, which do not have ribosomes, but including, for example, platelets, which maintain the ability to synthesize proteins thanks to a small pool of ribosomes inherited from their precursor megakaryocytes [20]. Translation of the SARS-CoV-2 S protein mRNA into active S proteins could therefore in principle occur anywhere in the body, as also suggested by the ability of the lipid nanoparticle–mRNA formulations of both BioNTech–Pfizer and Moderna preparations to reach virtually any organ and tissue in preclinical biodistribution studies in rodents [1,2].

Prodrugs lack the pharmacological activity of their active moieties; however, they may contribute to the overall safety and toxicity profile of the drug product, and therefore their evaluation is usually included in the overall assessment of new preparations [19].

### 4.1. Pharmacology of the SARS-CoV-2 S Protein mRNA

The SARS-CoV-2 S protein mRNA contained in COVID-19 mRNA vaccines has a complex pharmacology. The topic has recently been the subject of an excellent review that focused on the possibility that mRNA vaccines eventually alter the genomes of human cells through retroposition and integration [21]. The author concludes by asking for experiments that specifically address the issue of genome integration safety [21]. Indeed, integration of DNA copies of SARS-CoV-2 sequences into the genome of infected human cells has been recently described, and chimeric transcripts were detected in patient-derived tissues [22], a finding which raised significant controversy and debate [23,24,25]. Nonetheless, recently, a case report was published showing the persistence of residual SARS-CoV-2 RNA and antigens in the appendix, skin and breast tissues of two patients who exhibited long COVID-19 symptoms 163 and 426 days after symptom onset [26]. Remarkably, intracellular reverse transcription of the BioNTech–Pfizer COVID-19 mRNA vaccine has been shown in vitro in the human liver cell line Huh7 [27], although its relevance still awaits to be assessed in in vivo models [28]. Nevertheless, the issue deserves careful consideration in view, for example, of the presence of vaccine mRNA and vaccine-induced S protein in axillary lymph nodes up to 60 days after both mRNA-1273 or BNT162b2 COVID-19 vaccines [9] as well as the occurrence of the S protein in circulating monocytes of vaccinated subjects with PASC-like symptoms many months after vaccination [11].

### 4.2. Pharmacology of the S Protein

Translation of SARS-CoV-2 S protein mRNA contained in COVID-19 mRNA vaccines results in the endogenous production of the S protein. The main molecular targets of the S protein are summarized in Table 1.

#### 4.2.1. Angiotensin-Converting Enzyme 2 (ACE2)

ACE2 is a peptidase that metabolizes the vasoconstrictor angiotensin II into the vasodilator angiotensin (1–7). ACE2 exists in soluble as well as membrane-bound forms, the latter being expressed in many organs including the gastrointestinal tract, the kidney and the heart. Cell entry of SARS-CoV depends on S protein binding to ACE2 [35]. Vaccine-induced S protein binding to ACE2 as a potential trigger for platelet aggregation, thrombosis and inflammation, as well as for hypertension and other cardiovascular disease, has been the subject of insightful reviews [36,37].

#### 4.2.2. CD147

CD147 is a transmembrane glycoprotein of the immunoglobulin superfamily which has been suggested to mediate SARS-CoV-2 entering host cells by endocytosis [31]. SARS-CoV-2 S protein–CD147 interaction disrupts human cardiac pericyte function through stimulation of the phosphorylation/activation of the extracellular signal-regulated kinase 1/2 (ERK1/2), thus representing a potential additional mechanism of S protein-induced microvascular damage [38]. The S protein disrupted cardiac pericyte function at the concentration of 1 μg/mL, and thus in the nM concentration range, in agreement with the reported S protein–CD147 affinity (Table 1). During COVID-19, S protein–CD147 interaction has been involved in damage to cardiomyocytes [39] as well as in alteration of erythrocyte morphology, eventually resulting in hyperviscosity syndrome [40] and hemolytic anemia [41], and even possibly in neurodegenerative processes [42]. The relevance of these findings for the safety of vaccine-induced S protein has never been considered.

#### 4.2.3. TLRs

Toll-like receptors (TLRs) are a group of transmembrane proteins, belonging to the pattern recognition receptor family, which recognize specific pathogen-associated molecular patterns and initiate intracellular signaling events, resulting in the secretion of type I interferon, inflammatory cytokines and chemokines [43]. Several TLRs may be implicated in COVID-19 and in the effects of COVID-19 vaccines; however, the strongest evidence provisionally points to TLR4 and TLR2.

Molecular docking studies have shown that the S protein trimer directly binds TLR4 [32]. S protein–TLR4 binding activates TLR4 signaling, possibly resulting in increased membrane expression of ACE2 and subsequent enhancement of SARS-CoV-2 entry, as well as to direct enhancement of the excessive inflammatory response involved in lung damage, myocarditis and multiple-organ injury [44].

TLR2 has been implicated in the increased production of inflammatory cytokines and chemokines in human and mouse macrophages and lung epithelial cells after in vitro exposure to the S protein [33]. Remarkably, epithelial cells expressing S protein intracellularly are noninflammatory, but elicit an inflammatory response in macrophages when co-cultured. The involvement of TLR2 is indicated in these experiments by the activation of the NF-κB pathway and by the lack of response to the S protein in Tlr2-deficient macrophages [33].

The role of S protein binding to TLR4 or TLR2 in the effects of COVID-19 vaccines has never been investigated; however, at least one preprint study exists showing that vaccination of healthy subjects with the BioNTech–Pfizer COVID-19 mRNA vaccine reduced the response of innate immune cells to TLR4 and TLR7/8 ligands, while increasing fungi-induced cytokine responses [45]. Any clinical implications of these findings are presently unknown.

#### 4.2.4. ERα

By use of a commercial protein microarray methodology and surface plasmon resonance kinetic analyses, the SARS-CoV-2 S protein was recently shown to bind with high-affinity estrogen receptor alpha (ERα) [34]. The study, presently available as a preprint in bioRxiv and in PubMed Central, also includes in vitro experiments in cultured cell lines. Of particular relevance, the S protein at the concentration of 10 ng/mL (about 0.13 nM) increased the proliferation of the breast cancer cell line MCF-7 through the activation of ER, as suggested by the ability of raloxifene, a potent and selective ER modulator, to block this effect [34]. The clinical relevance of S protein–ERα interaction is suggested by postmortem experiments in lung tissues from SARS-CoV-2-infected rodents and humans showing increased ERα expression and ERα-S protein colocalization in alveolar macrophages [34]. The possibility that the SARS-CoV-2 S protein is endowed with estrogen-like properties provides novel clues for the interpretation of menstrual irregularities commonly observed after COVID-19 vaccination [46]. On the other hand, estrogenic compounds are established factors in the initiation and progression of breast cancer [47]; therefore, the high affinity of the S protein for ERα should encourage in-depth investigations about any possible influence of SARS-CoV-2 infection as well as of COVID-19 vaccination on breast cancer.

#### 4.2.5. The SARS-CoV-2 S Protein Alone Directly Affects Human Cells

The SARS-CoV-2 S protein exerts direct effects on human cells even in the absence of other viral components [48]. For example, treatment of human pulmonary artery smooth muscle cells or endothelial cells with 10 ng/mL (0.13 nM) SARS-CoV-2 S protein S1 subunit activates cell growth signaling, an effect which is consistent with the pulmonary vascular wall thickening of patients who died of COVID-19 [49]. Another study showed that transient transfection of the human lung alveolar epithelial cell line A549 or of the human liver epithelial cell line Huh7.5 with SARS-CoV-2 S protein results in increased activation of proinflammatory NF-κB and AP-1 transcription factors, and of p38 and ERK mitogen-activated protein kinases, finally resulting in increased release of interleukin (IL)-6, through downregulation of ACE2 protein expression and subsequently activation of angiotensin II type 1 receptors [50]. Cell lines transiently transfected with the SARS-CoV-2 S protein may well reflect the consequences of vaccine-induced endogenous production of the S protein, while in vitro experiments with the SARS-CoV-2 S protein may predict the effects of the free-floating protein in plasma and extracellular fluids.

In summary, both the SARS-CoV-2 S protein mRNA and the S protein itself exhibit a complex pharmacological profile with potential toxicological issues. None of these issues, however, were taken into consideration in the studies that led to the marketing authorization [1,2], precisely because, first of all, from a regulatory point of view, these products were treated as conventional vaccines.

## 5. COVID-19 mRNA Vaccines as Pharmaceutical Drugs: Regulatory Implications

Defining the nature of COVID-19 mRNA vaccines is not just a matter of confrontation of scientific opinions. Regulatory agencies have defined these products a priori as conventional vaccines, and as a consequence, they referred to the applicable product guidelines [51,52] when it came to evaluating applications for COVID-19 vaccines for subsequent marketing authorization. Guidelines on evaluation of vaccines obviously focus on their ability to stimulate the immune system, since they are intended to deal with just antigens and antigen preparations with no other expected activities, while guidelines on evaluation of pharmaceutical drugs require a global assessment of pharmacodynamics, pharmacokinetics and clinical pharmacology.

### 5.1. Preclinical Assessment

According to the WHO guidelines on nonclinical evaluation of vaccines [51], “*A pharmacodynamic study for a vaccine product is generally conducted to evaluate the immunogenicity. However, a pharmacodynamic study may also extend to include the pharmacology of an adjuvant*” (page 43), “*Toxicity studies should address the potential of the product for causing local inflammatory reactions, and possible effects on the draining lymph nodes, systemic toxicity and on the immune system*” (page 47), “*Genotoxicity studies are normally not needed for the final vaccine formulation*” (page 50), and “*Pharmacokinetic studies (e.g., for determining serum or tissue concentrations of vaccine components) are normally not needed*” (page 51).

For comparison, the International Council for Harmonisation of Technical Requirements for Pharmaceuticals for Human Use (ICH) guidelines, nonclinical studies for the conduct of human clinical trials and marketing authorization for pharmaceuticals [53], recommend “*primary pharmacodynamic studies * (in vivo *and/or* in vitro*) […] intended to investigate the mode of action and/or effects of a substance in relation to its desired therapeutic target*” as well as “*safety pharmacology studies [including] the assessment of effects on cardiovascular, central nervous and respiratory systems*” (page 5). Toxicokinetic and pharmacokinetic studies (including absorption, distribution, metabolism and excretion), as well as acute and repeated-dose toxicity, are also required, and genotoxicity studies are mandatory either as assays for gene mutation (to support single-dose clinical development trials) or as assays for chromosomal damage in mammalian systems (for multiple-dose clinical development trials).

The complex pharmacological profile of both the SARS-CoV-2 S protein mRNA contained in COVID-19 vaccines and the resulting S protein, together with the evidence of their systemic disposition, would better fit with the comprehensive assessment recommended for pharmaceuticals, in comparison to the assessment focused on immunogenic properties required for conventional vaccines. Unfortunately, the latter was chosen as a reference for COVID-19 mRNA vaccines, as explicitly indicated in the EMA assessment reports [1,2]. As a consequence, preclinical assessment of these products did not include any secondary pharmacodynamic studies, safety pharmacology studies, pharmacodynamic drug interaction studies, traditional pharmacokinetic or biodistribution studies and/or genotoxicity studies, and all these omissions are defined as acceptable/agreeable by the EMA Committee for Medicinal Products for Human Use (CHMP). Specifically, the EMA assessment reports never mention any pharmacological and functional properties of the SARS-CoV-2 S protein mRNA and/or of the S protein that are described above in Section 4.

### 5.2. Clinical Assessment

The WHO guidelines on clinical evaluation of vaccines [52] are consistent with the WHO nonclinical guidelines and deal first of all with assessment of the immunogenicity and of the resulting protective efficacy of candidate vaccines. One of the most remarkable differences in the ICH guidelines for pharmaceuticals is that “*The collection of data on routine laboratory tests (hematology, chemistry and urine analysis) is not necessary in many clinical trials of vaccines*” (page 564). Indeed, clinical laboratory tests were performed only in the small phase I part of the trial programs, in which just a few dozen participants were enrolled. Even such a small sample was enough to identify several laboratory changes: for example, the BioNTech–Pfizer phase I study recorded between 8.3% and 33.3% grade 3 decreases in the lymphocyte count in each dose group and grade 2 neutropenia in two other participants [54]. Despite such findings, no clinical laboratory evaluations were subsequently included in the phase III trial [55].

The importance of clinical laboratory evaluations in the overall safety assessment of any new pharmaceuticals is well-explained in the ICH guidelines. According to the Common Technical Document (CTD) Efficacy (M4E) [56], describing the structure and format of the clinical data in a new drug application and detailed reporting of changes in patterns of laboratory tests, including “*hematology, clinical chemistry, urinalysis and other data as appropriate*”, are crucial for the interpretation of observed adverse events. In particular, the CTD clearly recommends considering “*laboratory findings reflecting actual or possible serious medical effects*”, since “*examination of which subjects experience extreme laboratory value abnormalities (“outliers”) may be useful in identifying subgroups of individuals who are at particular risk for certain adverse events*” (Section 2.7.4.2.1 of Analysis of Adverse Events).

Spontaneous studies exist, suggesting the usefulness of laboratory tests after vaccination to better characterize COVID-19-vaccine-induced effects, eventually identifying subjects potentially at risk for the development of clinically relevant adverse reactions. For example, a study in 281 vaccinated subjects, including 143 vaccinated with the BioNTech–Pfizer vaccine, showed that 6.8% (5.6% of those who received the BioNTech–Pfizer vaccine) tested positive for anti-PF4/polyanion antibodies postvaccination [57]. Anti-PF4/polyanion antibodies are associated with vaccine-induced immune thrombotic thrombocytopenia (VITT), and although in this study antibody levels were never high enough to induce platelet aggregation, subjects with anti-PF4/polyanion antibodies could be considered a subgroup at risk for VITT, and consequently were offered long-term monitoring and eventually a timely treatment. Another study, considering 566 patients followed in a cardiology clinic for cardiovascular risk assessment, documented an increase in various inflammatory markers known to predict the 5-year risk of acute coronary syndromes following BioNTech–Pfizer or Moderna mRNA vaccines [58], suggesting the opportunity to assess the predictive value of these markers for the identification of subjects at risk for cardiovascular events. As a further example, markers of platelet activation and increased proinflammatory cytokines were found in the blood of 50 patients who experienced post-acute sequelae of COVID-19 (PASC)-like symptoms following SARS-CoV-2 vaccines, but not in 10 healthy subjects or in 35 vaccinated subjects without PASC-like symptoms [11]. Whether this laboratory pattern represents a biomarker signature for some kind of adverse events following COVID-19 vaccination would deserve consideration.

Failure to include clinical laboratory evaluations in the clinical assessment of COVID-19 vaccines led many governments and institutions to take this “absence of evidence as evidence of absence”, and consequently to not recommend (and in some cases even to advise against) performing any kind of examination before or after vaccinations. A prominent example in Italy is the position adopted by the National Federation of Orders of Surgeons and Dentists (FNOMCeO) [59].

### 5.3. Post-Marketing Safety Assessment

COVID-19 mRNA vaccines have been included in the EMA additional monitoring list as they contain new active substances not contained in any previous authorized medicinal product, and they are approved under a conditional marketing authorization [1,2]. However, although the risk management plans for these products include a few additional specific studies to address, e.g., myocarditis/pericarditis, safety in pediatric subjects, and in pregnancy, most of the post-marketing safety assessment is essentially based on activities related to the receipt and review of individual spontaneous adverse event reports sent by physicians, other health care workers and by the general public [60,61].

This approach suffers from two major limitations. The first one is the well-known underreporting, which in “normal” times has been estimated in the order of 82–98% of all adverse events, and even higher for serious/severe events [62]. In the case of COVID-19 vaccines, such underreporting may be, however, even more dramatic and extreme. Let us look at the latest Italian report on the surveillance of COVID-19 vaccines [63], which summarizes about a year and a half of monitoring. The report apparently includes 93% spontaneous reporting and an additional 7% reports coming from “active pharmacovigilance” unspecified studies, possibly the additional studies mentioned by the EMA in its abovementioned risk management plans. The key issue is, however, that, while AIFA reports about 100 suspect adverse events per 100,000 doses administered, over the same period, the USA active surveillance system v-safe recorded about 68,600 local reactions and 52,700 systemic reactions per 100,000 doses after the first dose, and 71,700 local reactions and 70,800 systemic reactions per 100,000 doses after the second dose, which is 70,300 per 100,000 local reactions and 61,750 per 100,000 systemic reactions [64]. Taking the v-safe data as a standard reference, the AIFA spontaneous reporting system suffers from a rate of about 99.92%; that is, less than 1 in 1000 adverse events are reported to the system. Underreporting is even higher in the case of severe adverse events (i.e., those events which require intervention to prevent permanent impairment or damage, result in disability or permanent damage, require or prolong hospitalization, result in congenital anomalies/birth defects, result in death), as AIFA reports 3.8 severe events per 100,000 doses and v-safe reports 17,700 per 100,000 doses, that is, 4650 reports in v-safe per each report in AIFA. Thus, despite the EMA additional monitoring, taking Italy as a reference (and indeed it is, since it is constantly among the countries with the highest absolute number of COVID-19-vaccine-related reports included in the EudraVigilance system—https://www.adrreports.eu/—accessed on 8 September 2022), the pharmacovigilance systems for these products are likely missing more than 999 adverse events of any severity per 1000 events, and more than 4998 severe adverse events per 5000 events.

Underreporting for post-marketing surveillance of COVID-19 vaccines is, however, a minor problem in comparison to the consequences of considering COVID-19 vaccines as conventional vaccines and not as pharmaceutical drugs. Any spontaneous report of suspect adverse events must indeed undergo a formal case causality assessment, which is, however, based on profoundly different procedures for conventional vaccines and for pharmaceutical drugs. As stated in the Uppsala Monitoring Center guidelines for standardized case causality assessment for pharmaceutical drugs, “*Since pharmacovigilance is particularly concerned with the detection of unknown and unexpected adverse reactions, other criteria such as previous knowledge and statistical chance play a less prominent role in the system*” [65] On the contrary, the WHO guidelines for causality assessment of an adverse event following immunization (AEFI) state that “*two critical questions in the revised WHO causality algorithm, namely: “Is there evidence in published peer reviewed literature that this vaccine may cause such an event if administered correctly?” and “In this patient, did the event occur within a plausible time window after vaccine administration?”. It is important to consider the background rates for the occurrence of an event of interest and then after a population has received vaccine, determine if the observed rate of that event is in excess of the background rates*” [66]. Comparison of these two introductory sentences clearly shows how causality assessment for pharmaceutical drugs expressly aims at identifying any “*unknown and unexpected*” adverse reaction, and to this end, it is clearly stated that “*previous knowledge and statistical chance*” are not key issues. On the other hand, causality assessment for conventional vaccines considers “*evidence in published peer reviewed literature*” as a prerequisite and requires that “*the observed rate of that event is in excess of the background rates*”. The prerequisite required for vaccines is a kind of catch-22 (from the title of a novel (1961) by the U.S. writer J. Heller (1923–1999)), i.e., an adverse event could be related to vaccines provided that the scientific literature will publish similar vaccine-related events, which, however, will hardly occur until the relationship is acknowledged. Indeed, in general, the causality assessment guidelines for conventional vaccines have been extensively criticized because from the onset of the evaluation, they recommend considering all possible “other causes” that might explain the adverse event and thus exclude the role of the vaccine. Subsequently, even if there was biological plausibility and temporal compatibility for a causal association between the vaccine and the event, the guidelines recommend looking for any possible evidence that the vaccine could not have caused that event. WHO guidelines for vaccines are therefore very strict and tend to exclude vaccines [67,68]. The consequences of this methodological approach will be illustrated and discussed taking COVID-19-vaccine-associated myopericarditis as an example.

#### Myopericarditis as Case Studies

Up to June 2021, the U.S.A. Centers for Disease Control and Prevention deemed the association between COVID-19 mRNA vaccines and myocarditis as possible, however, at an incidence of just about 0.5 cases per 100,000 based on spontaneous reporting [69]. In August 2021, however, data from a network of 40 hospitals in Washington, Oregon, Montana and Los Angeles County, California, showed that emergency department or inpatient encounters with diagnoses of myocarditis or pericarditis after COVID-19 vaccines could be as high as 1 case per 100,000 for myocarditis and 1.8 for pericarditis [70], thus providing direct evidence for underreporting to the CDC pharmacovigilance system, even if the study did not address cases outside care settings and cases of subclinical myocarditis or pericarditis. Many other studies subsequently addressed the issue by querying large health care databases and reporting even higher figures, e.g., [71,72]. Some studies also aimed at comparing COVID-19-vaccines- and COVID-19-associated risks: a major example is based on data from the English National Immunisation (NIMS) database of COVID-19 vaccination [73]. The authors linked data in the NIMS database, at the individual patient level, to national data for mortality, hospital admissions and SARS-CoV-2 infection, finding, for example, that in the general population, the BioNTech–Pfizer vaccine, the Moderna vaccine and SARS-CoV-2 infection were associated with, respectively 1, 16 and 40 cases of myocarditis per 1,000,000 exposed, and that in people aged under 40 years, there were 5, 23 and 10, respectively, excess cases per 1,000,000 exposed [73]. Remarkably, however, assessment of risk associated with COVID-19 vaccines usually implies the preliminary exclusion of people with previous myopericarditis as well as people with a previous case of COVID-19, in implicit agreement with the WHO AEFI guidelines that favor the existence of alternative causes, thus excluding any role for vaccines [66], and in contrast with the Uppsala Monitoring Center guidelines for pharmaceutical drugs, which rather consider previous disease and/or comorbidities just as potential contributing causes [65]. Indeed, a study based on data of the French National Health Data System (SNDS), which covers more than 99% of the French population, recently showed that the odds ratio for COVID-19-mRNA-vaccines-associated myocarditis or pericarditis was on average 6.3 and 3.9, respectively, with a history of SARS-CoV-2 infection in the previous 30 days and up to 140 and 250 for those with a history of myocarditis or pericarditis [74].

Another consequence of the recommendations provided by the WHO AEFI guidelines is the need to identify in advance a time window of increased risk based on some kind of biological plausibility [66]. Usually, the majority of adverse reactions to conventional vaccines are expected to occur because of excessive or biased inflammatory and immune responses; thus, time windows are narrow. In most of the studies on COVID-19 vaccines, including the examples discussed above, time windows are usually set at two weeks after each dose or in some cases at 4–6 weeks following completion of the vaccination cycle. Such a narrow time window is also adopted by regulatory bodies, such as, for example, the Italian Medicines Agency (Agenzia italiana del farmaco, AIFA), which openly declares that a time window of just two weeks applies even for serious and fatal events [75]. An approximate estimate of the consequences of this restrictive approach is provided by a recent study in the national health care databases of the U.S.A. Department of Veterans Affairs assessing the frequency of a set of serious adverse events over a period of 38 weeks following COVID-19 mRNA vaccines [76]. Results show the occurrence of 1512.9 events per 10,000 with the BioNTech–Pfizer product and 1422.3 events per 10,000 with the Moderna product, with an excess in the BioNTech–Pfizer group of 10.9 ischemic strokes, 14.8 myocardial infarctions, 11.3 other thromboembolic events and 17.1 kidney injuries, which makes a total of 53.1 serious adverse events per 10,000 vaccinated subjects, which is about 1 in 200. These findings are somewhat in agreement with a secondary analysis of serious adverse events in the placebo-controlled, phase III randomized clinical trials of BioNTech–Pfizer and Moderna mRNA COVID-19 vaccines in adults, focused on Brighton Collaboration adverse events of special interest [77]. BioNTech–Pfizer and Moderna mRNA vaccines were associated with an absolute increase in risk for serious adverse events of special interest of 12.5 per 10,000, based on a median follow up of about 2 months, for comparison with a risk for a reduction in COVID-19 hospitalization relative to the placebo group of 2.3 (BioNTech–Pfizer) and 6.4 (Moderna) per 10,000 participants [77].

In summary, founding the post-marketing safety assessment of COVID-19 mRNA vaccines just on spontaneous adverse event reporting systems is likely biased by an unprecedented level of underreporting. In addition, using the WHO AEFI guidelines for causality assessment of reports likely leads to neglecting a large part of the relevant information, mainly due to the imposition of too narrow time windows after vaccinations, as well as the biased attitude of identifying previous disease and/or comorbidities as alternative explanations rather than as just potential contributing causes [66]. In particular, concerning time windows, as already discussed in Section 2, growing evidence shows that vaccine-derived S protein persists for several months after vaccinations, in particular in subjects with adverse events following vaccination [8,11,12]. In particular, the occurrence of vaccine-derived S protein in endomyocardial biopsies of patients with myocarditis up to nearly two months following COVID-19 vaccination is paradigmatic [10].

Finally, a recent study systematically looking for cardiovascular effects in 301 students aged 13–18 years who received the second dose of the BioNTech–Pfizer COVID-19 mRNA vaccine provides excellent evidence of what can be achieved through an intensive safety monitoring approach: seven participants (2.3%) exhibited at least one elevated cardiac biomarker or positive lab assessments, one participant had myopericarditis, two participants had suspected pericarditis, and four participants had suspected subclinical myocarditis [78]. These findings indicate that it is at least unrealistic to compare the frequency of adverse effects following vaccination in comparison to SARS-CoV-2 infection based on currently available information on vaccines.

## 6. Conclusions

Since translation of the mRNA occurs potentially and—most importantly—unpredictably in any tissues and organs, and it can be easily hypothesized that inappropriate production in vulnerable tissues may represent a major risk factor for local tissue damage, leading to myocarditis, central and peripheral neuropathies, vasculopathies, myopathies, endocrinopathies and other disease, depending on the location and amount of S protein expression (or of local distribution from general circulation). In addition, it is well-known that distinct tissues widely differ in the efficiency of protein synthesis, but no one so far has assessed whether and to what extent this might be relevant for the efficacy and safety of mRNA vaccines [79]. For instance, metabolic labeling techniques have been developed to measure the flux rate of protein synthesis in human muscle for conditions of altered muscle mass and function [80], and it might be of interest to develop similar approaches to predict the expected production of S protein following COVID-19 vaccination. Knowledge of vaccine-induced S protein disposition could also greatly help in defining the best individual vaccination regimen, in terms of dose as well as of time interval between doses. We recently estimated a likely S protein clearance (CL) based on experimental data from other peptides [15]; however, it would be easy to directly measure S protein CL in humans by means of simple and conventional approaches already established for other drugs. Detailed knowledge of biodistribution and disposition of vaccine-induced S protein would allow integration of its pharmacokinetics and pharmacodynamics, thus allowing the description of the time course of its effects in individual subjects, likely leading to, e.g., individualization of dose and administration as well as timely identification of subjects at risk for major adverse effects. The pharmacogenetics of sensitive mechanisms and targets such as the ribosomal machinery involved in RNA translation to S protein, as well as polymorphisms of the ACE2 receptor [81], should be incorporated into the model.

Unfortunately, at present we have almost none of the information necessary to address and manage all these aspects, and to use COVID-19 mRNA vaccines in a conscious, targeted and rational way. The present opinion paper was written with the aim of providing guidance to prioritize studies on these products, assessing their pharmaco-toxicological profile, their disposition, clinical pharmacology and safety, by using appropriate approaches developed for pharmaceutical drugs. We discussed the pharmacology and functional profile of SARS-CoV-2 S protein mRNA included in vaccines and of vaccine-derived S protein; however, we are well-aware that the pharmacological and clinical assessment of these drugs must take also into account their pharmaceutical preparation, which is based on the mRNA encapsulation into newly developed lipid nanoparticles (LNPs). LNPs likely contribute to reactogenicity and immunogenicity of these products, and their proinflammatory potential is a matter of concern [82]. In addition, LNPs are crucial for mRNA stability, and it is remarkable that the structure of mRNA encapsulated into LNPs largely remains to be established [83]. Whether and to what extent such uncertainties impact on mRNA stability and on the overall pharmaceutical quality of these products is another issue that must be addressed in order to improve their use [84,85].

Another key issue with major implications for the quality and safety of COVID-19 mRNA vaccines regards the authorization of the so-called “adapted” vaccines, which are expected to protect against emerging SARS-CoV-2 variants [86,87]. Such products are being authorized following the results of small immunogenicity studies focusing on levels and in vitro neutralizing activity of circulating antibodies following vaccination [88]. Considering, however, the issues discussed above concerning the pharmacological profile of the SARS-CoV-2 S protein mRNA as well as the resulting pharmaceutical products, the risks involved in deeming the products safe as “*it is just a matter of another mRNA sequence*” should be evident.

In summary, we have highlighted the pitfalls of having considered until now COVID-19 mRNA vaccines as just conventional vaccines, and we have indicated the preclinical, clinical and post-marketing safety assessments that are most urgently needed. COVID-19 mRNA vaccines are actually pharmaceutical drugs, and consequently their pharmacokinetics and pharmacodynamics, and possibly also their pharmacogenetics, must be properly characterized [89] to provide a solid background of knowledge for their rational and targeted use, thus stopping “playing dice” with these products due to the misbelief that the same vaccine at the same dose is good for everyone, and that adverse effects occur just by chance. A correct, rigorous and complete evaluation of COVID-19 mRNA vaccines will be of critical importance for reassuring the public about their safe and effective use, eventually overcoming vaccine hesitancy.

## Figures and Tables

**Table 1:** Molecular targets of SARS-CoV-2 S protein.

Target	Active Concentrations	Reference
ACE2 ^1^	1.58–120 nM (K_D_^2^)	[29,30]
CD147	185 nM (K_D_^2^)	[31]
TLR4 ^3^	300 nM (K_D_^2^)	[32]
TLR2 ^4^	500 ng/mL (6.5 nM) ^5^	[33]
Erα ^6^	9.7 nM (K_D_^2^)	[34]

^1^ angiotensin-converting enzyme 2; ^2^ K_D_ = equilibrium dissociation constant, a constant which measures the propensity for the bound ligand–target complex to dissociate to free ligand and target, and corresponds to the ligand concentration which is necessary to bind 50% of the available target; ^3^ Toll-like receptor 4; ^4^ Toll-like receptor 2; ^5^ active concentration in functional experiments; ^6^ estrogen receptor alpha.

## Data Availability

Not applicable.
